# Neutrophil gelatinase-associated lipocalin (NGAL) fails as an early predictor of contrast induced nephropathy in chronic kidney disease (ANTI-CI-AKI study)

**DOI:** 10.1038/srep41300

**Published:** 2017-01-27

**Authors:** Werner Ribitsch, Gernot Schilcher, Franz Quehenberger, Stefan Pilz, Rupert H. Portugaller, Martini Truschnig-Wilders, Robert Zweiker, Marianne Brodmann, Philipp Stiegler, Alexander R. Rosenkranz, John W. Pickering, Joerg H. Horina

**Affiliations:** 1 Clinical Division of Nephrology, Department of Internal Medicine, Medical University of Graz (MUG), Austria; 2 Intensive Care Unit, Department of Internal Medicine, MUG, Austria; 3 Institute for Medical Informatics, Statistics and Documentation, Medical University of Graz, Austria; 4 Division of Endocrinology and Metabolism, Department of Internal Medicine, Medical University of Graz, Austria; 5 Department of Vascular and Interventional Radiology, University Clinic of Radiology, Medical University of Graz, Austria; 6 Clinical Institute for Medical and Chemical Laboratory Diagnostics, Medical University of Graz, Austria; 7 Division of Cardiology, Department of Internal Medicine, Medical University of Graz, Austria; 8 Division of Angiology, Department of Internal Medicine, Medical University of Graz, Austria; 9 Division of Transplantation Surgery, Medical University of Graz, Austria; 10 Department of Medicine, University of Otago Christchurch and Emergency Medicine Department, Christchurch Hospital, Christchurch, New Zealand

## Abstract

The aim of the study was to evaluate the diagnostic accuracy of urinary neutrophil gelatinase- associated lipocalin (uNGAL) in patients with chronic kidney disease (CKD) as an early biomarker for contrast induced acute kidney injury (CI-AKI) and to investigate whether patients with an uNGAL increase might benefit from an additional intravenous volume expansion with regard to CI-AKI-incidence. We performed a prospective randomized controlled trial in 617 CKD-patients undergoing intra-arterial angiography. Urinary NGAL was measured the day before and 4–6hrs after angiography. In the event of a significant rise of uNGAL patients were randomized either into Group A, who received intravenous saline post procedure or Group B, who did not receive post-procedural i.v. fluids. Ten patients (1.62%) exhibited a significant rise of uNGAL after angiography and were randomized of whom one developed a CI-AKI. In the entire cohort the incidence of CI-AKI was 9.4% (58 patients) resulting in a specificity of 98.4% (95% CI: 97.0–99.3%) and a sensitivity of 1.72% (95% CI: 0.044–9.2%) of uNGAL for the diagnosis of CI-AKI. In this study uNGAL failed to predict CI-AKI and was an inadequate triage tool to guide an early intervention strategy to prevent CI-AKI. Clinical Trial Registration: URL: http://www.clinicaltrials.gov. Unique identifier: NCT01292317.

Contrast induced acute kidney injury (CI-AKI) also called contrast induced nephropathy (CIN) represents a serious complication of radiocontrast procedures and has consistently been associated with adverse clinical outcomes^1^,^2^. Chronic kidney disease (CKD) alone or coupled to other risk factors such as diabetes or heart failure significantly increases the incidence of CI-AKI to 25–45%^3^,^4^. Although plasma creatinine only poorly reflects early changes in glomerular filtration it is still the mainstay of all current definitions of acute kidney injury (AKI) and CI-AKI^5^. The use of plasma creatinine for the detection of AKI fosters a therapeutic intervention not before 24 hours following the insult to the kidney. Hence, considerable effort has been put into the search for new biomarkers as early indicators of AKI^6^. One of the promising candidate biomarkers is neutrophil gelatinase - associated lipocalin (NGAL), a 25 kDa siderophore binding protein^7^. In response to a renal tubular injury the monomeric form of NGAL is highly expressed in the kidney followed by a rapid increase of NGAL-levels in plasma and urine^8^,^9^. Clinical trials in adults reported a convincing performance of NGAL to diagnose AKI as early as 4–8 hours after a renal injury, far earlier than by an increase in creatinine concentrations^4^,^10^,^11^. However, it has not been evaluated yet if a therapeutic intervention prompted by an early NGAL-rise can prevent further kidney damage and/or a subsequent deterioration of glomerular filtration. Given the reported high incidence of CI-AKI in patients with CKD, we hypothesized that administration of intravenous saline after an early post procedural rise of uNGAL would prevent further kidney damage reducing the incidence of CI-AKI^12^. We further aimed to quantify the diagnostic accuracy of uNGAL as an early biomarker of CI-AKI in patients with chronic kidney disease (CKD).

## Materials and Methods

### Study population

We conducted a single-center, open-label, prospective randomized trial on patients with CKD who either underwent a percutaneous coronary intervention (PCI) or a percutaneous transluminal angiography/angioplasty (PTA) of peripheral arteries, renal arteries or carotids (clinicaltrials.gov: NCT01292317, registered February 8, 2011). No patients undergoing emergency procedures were included. Briefly, inclusion criteria were age >18 years, stable CKD with an estimated glomerular filtration rate (eGFR) <70 mL/min per 1.73 m^2^ which was confirmed by checking the institutional medical record system and the laboratory values from out-of hospital testing provided by the patients, and the need for intra-arterial angiography/angioplasty. Criteria for exclusion were evidence of acute renal failure according to the Acute Kidney Injury Network (AKIN) criteria^13^, dependence on dialysis, rhabdomyolysis, intolerance of volume expansion, acute life threatening conditions, pregnancy and administration of iodinated contrast media within 7 days prior to intervention. The study was conducted in accordance with the Declaration of Helsinki at the Medical University of Graz and was approved by the Institutional Review Board of the Medical University of Graz (IRB00002556, Study Registration Number: 21–278 ex 09/10). All patients gave their written informed consent prior to enrollment in the study.

### Study design and conduct

The study design has been previously published in detail^12^. As a nephroprotective measure all study participants received 3–5 ml/kgBW 0.9% saline per hour for 3 hours prior to angiography (=1000 ml over 3 hours in a 70 kg person). Monomeric, non-ionic, low osmolar iomeprol (Iomeron 300^®^, Bracco Vienna, Austria) was exclusively used as contrast medium for all study subjects in doses adjusted for body weight and determined by the numbers and locations of angiograms. Urinary NGAL (uNGAL) was measured the day before (day-1) and 4–6 hours after CM-application (day 0). Urine samples were taken from spontaneously voided urine and immediately processed thereafter. For practical reasons we chose a time period instead of an exact point in time for the uNGAL-measurement. Patients whose uNGAL was elevated above a pre-determined threshold after angiography (day 0) were randomized into two groups (A and B) using an online-randomization tool (http://www.randomizer.at). Thresholds for randomization were an increase of uNGAL from <75 ng/ml at baseline (day-1) to >150 ng/ml post angiography (day 0) or a doubling of uNGAL if baseline values were >75 ng/ml. These increments of uNGAL were considered significant accounting for a standard deviation of uNGAL that is approximately twice its mean concentration in healthy subjects^14^,^15^. Patients allocated to Group A received 3–4 ml/kg/BW 0.9% saline intravenously for 6 hours post procedure, Group B did not receive any i.v. fluids.

#### Assays

Plasma creatinine was measured by the standard Jaffe colorimetric method, Cystatin C by a nephelometric immunoassay and the estimated glomerular filtration rate (eGFR) was calculated via the abbreviated Modification of Diet in Renal Disease (MDRD) study equation^16^. Midstream urinary NGAL (uNGAL) was measured the day before and 4–6 hours after CM-application by the ARCHITECT^®^ NGAL Test (Abbott Laboratories, Abbott Park, Illinois, US). Blood was drawn the day preceding angiography (day-1), the day after angiography (day 1) and the following day (day 2).

#### Outcomes

The primary outcome of the study was the development of CI-AKI by a ≥25% increase of plasma creatinine from baseline within 48 hours after angiography. This was the most commonly used definition and timing of CI-AKI at the time of creating the study protocol^1^,^17^. For a renal follow-up, plasma creatinine and eGFR were derived from the institutional medical record system one and three months after hospital discharge if available. In a secondary analysis we tested the sensitivity and specificity of uNGAL for CIN in the non-randomized cohort^12^.

### Statistical analysis

Descriptive statistics were given as median 25% percentile (Q_1_) - 75% percentile (Q_3_) or mean ± standard deviation. Associations (correlations) between continuous variables were tested by the Spearman rank correlation coefficient, associations between dichotomous variables and continuous or polytomous variables were tested by the Wilcoxon rank sum test or Kruskal-Wallis test. Associations between dichotomous variables were tested by Fisher’s exact test. Continuous variables were dichotomized at the median in order calculate descriptive statistics of uNGAL for the high and the low group. Risk factors for CI-AKI were assessed by univariate logistic regression. Differences between treatment arms were assessed by the Fisher’s exact test. Because of its skewed distributions uNGAL was transformed by the natural logarithm.

#### Sample size

Based on the reported high positive predictive value of NGAL for an early diagnosis of CI-AKI^18^,^19^,^20^ we considered the reduction in CI-AKI incidence by 20% in the hydration group A as clinically relevant. Therefore 108 patients per treatment arm are required at an alpha of 0.05 and power of 0.8 (two-sided, chi-squared test). Assuming a 20% CI-AKI incidence rate^21^,^22^,^23^ and a 10% drop out rate, then 1200 patients had to be included. It was pre-specified that if after inclusion of 240 patients less than 48 patients had been randomized, the NGAL criteria would be adapted to improve the recruitment rate to intervention. All statistical tests were two-sided. R 3.2.0 was used for calculations.

## Results

### Patients

**Figure 1:**
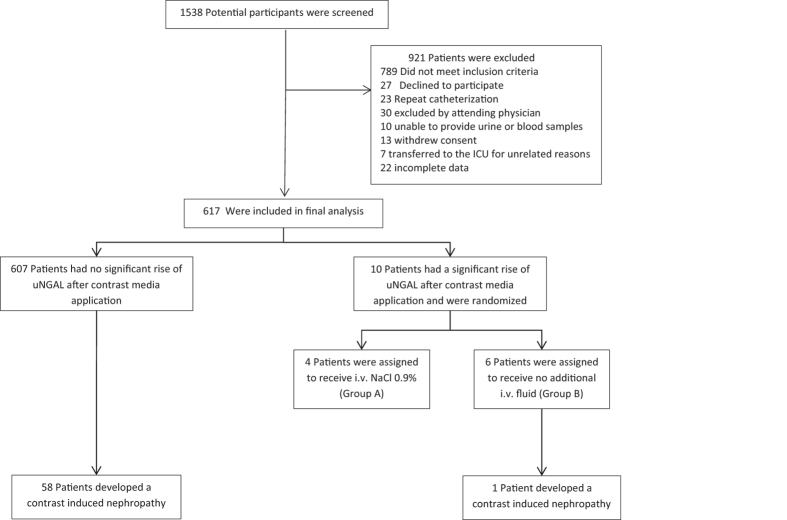
Enrollment, randomization and renal outcome during the ANTI-CI-AKI-study.

Between September 2010 and April 2012 1538 potential study participants were screened. After the screening process 921 patients had to be excluded: 789 did not meet the inclusion criteria, 27 declined to participate, 23 cases had to be excluded because patients underwent multiple angiographic procedures and 82 had miscellaneous reasons. (Fig. 1). This left 617 (287 female) patients (median age 74.0 years, range 37–91 years) who were recruited and finished the study in compliance with the study protocol. 39.9% of patients were diabetics, 85.9% had hypertension and 81.2% had an underlying heart disease which was defined as any history of ischemic heart disease, congestive heart failure, cardiac valvular defects or chronic cardiac arrhythmias. The mean plasma creatinine at study entry was 1.36 ± 0.41 mg/dl, and the mean eGFR was 48.7 ± 12.7 ml·min^−1^·1.73 m^−2^. 62.4% of participants underwent a PCI, 34.5% had a percutaneous transluminal angioplasty of peripheral arteries and 3.1% had a PTA of renal arteries or carotids, respectively (Table 1). The pre-procedural hydration according to our protocol was given to 97.2% of patients.

At the pre-planned interim assessment of the first 240 patients only two had been randomized. This unexpectedly low number meant that it was not possible to adjust the uNGAL criteria to sufficiently improve recruitment to meet the minimum needed number to test the primary hypothesis. This led to the decision not to change criteria for randomization and to continue to collect data to test the sensitivity and specificity of uNGAL for the diagnosis of CI-AKI in non-randomized patients. A sample size of 600 was considered sufficient for this.

### Contrast induced acute kidney injury *(Primary outcome)*

From all 617 patients included into our study, 58 patients (9.4%) developed a ≥25% increase of serum creatinine from baseline either 24 or 48 hours after angiography with a peak creatinine of 2.6 mg/dl on day 1 and 3.6 mg/dl on day 2. The incidence was 9.6% with the KDIGO-criteria of AKI^5^. By the RIFLE-classification of AKI stages, no patients were classified as RIFLE R (1.5fold increase of creatinine), 43 patients were classified as RIFLE I (a two-fold increase in creatinine) and 3 patients RIFLE F (a threefold increase of creatinine)^24^. No patients had oliguria.

**Figure 2:**
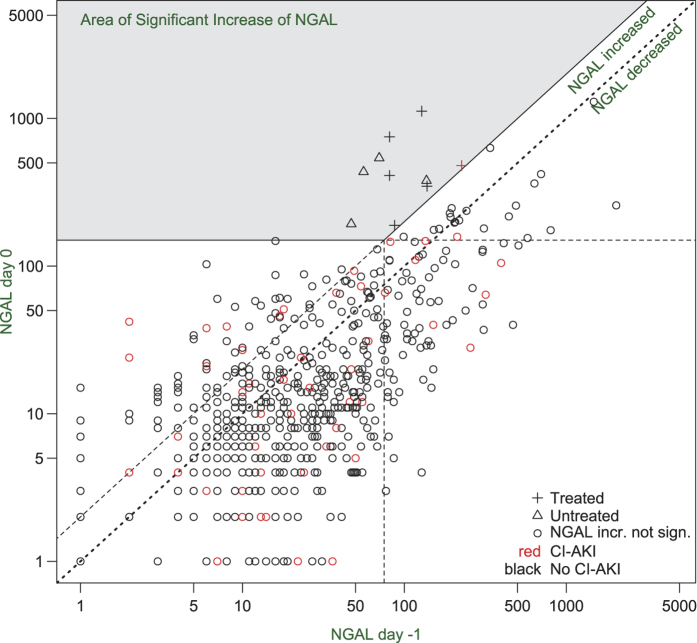
NGAL and CI-AKI diagnosis on a logarithmic scale. Black: Patients without CI-AKI; Red: Patients with CI-AKI; Shaded area: Patients with significant increase of NGAL. Diagonal dotted line: Patients without significant change of uNGAL. Diagonal dashed line: Patients with doubling of uNGAL after angiography. Area below dotted line: Patients with decrease of uNGAL.

Ten patients (9 female) showed a significant increase of uNGAL after angiography and were randomized. Six patients were assigned to group B, of whom one developed a CI-AKI, whereas no CI-AKI was observed in group A (p = 1.0, Fig. 2). In group B two adverse events (AE) occurred on day 0 and one severe adverse event (SAE) on day 1. In group A there was one AE and one SAE on day 0 and two AE on day 1. Detailed information about types and numbers of adverse events and adverse reactions is provided in Supplemental Tables 1 and 2 of the Supplementary appendix.

In the whole group patients with CI-AKI had lower creatinine and a higher eGFR at baseline. Apart from baseline creatinine and eGFR no other independent predictors of CI-AKI could be identified by logistic regression analysis (Table 2). Patients who developed a CI-AKI were significantly longer hospitalized (median two days, Q1–Q3 2–4 days) than patients without CI-AKI (median three days, Q1–Q3 2–7 days; p = 0.01). One month after angiography kidney function parameters of 363 patients could be derived from the institutional medical record system. Patients with CI-AKI had an eGFR of 56.50 ± 18.02 ml·min^−1^·1.73 m^−2^ and did not differ from patients without CI-AKI (eGFR 58.92 ± 24.92 ml·min^−1^·1.73 m^−2^; p = 0.76). Three months after hospital discharge kidney function parameters of 422 patients were available and remained stable in both groups (eGFR 55.25 ± 13.05 ml·min^−1^·1.73 m^−2^ versus 60.40 ± 22.72 ml·min^−1^·1.73 m^−2^; p = 0.22).

### Characteristics of uNGAL and prediction of contrast induced acute kidney injury *(Secondary analysis)*

**Figure 3:**
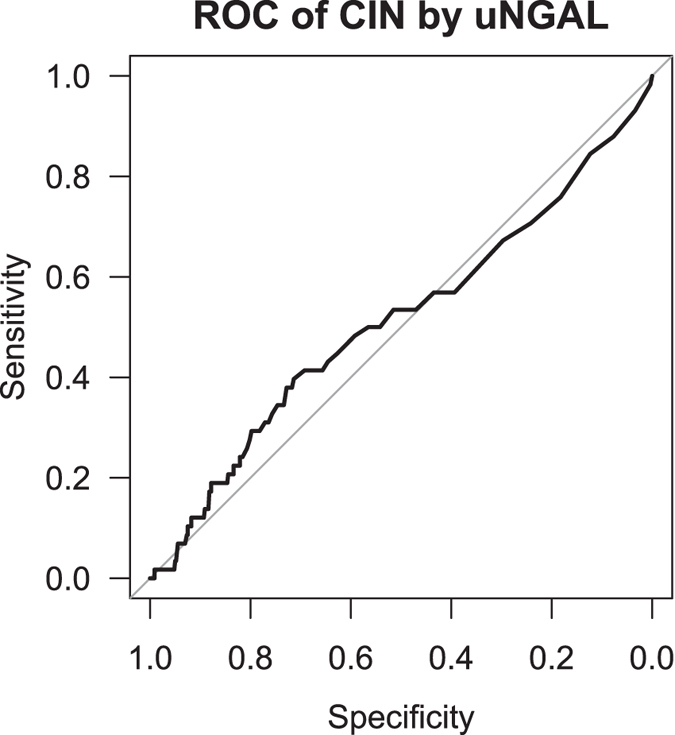
The receiver operating characteristic (ROC) curve for uNGAL to predict CI-AKI. The Area under the Curve was 0.51 (95% CI: 0.43–0.59).

In the whole group median uNGAL (Q1–Q_3_) was 19 (9–49) ng/ml at day-1. uNGAL was ≤75 ng/ml in 521 patients (84.4%). Baseline uNGAL was associated with female sex, diabetes, age, lower eGFR, Cystatin C, urine osmolality and proteinuria (Table 3). These associations applied for both absolute uNGAL and uNGAL normalized to creatinine (Table 3). In the whole group uNGAL decreased from median 19 (9–49) ng/ml at day-1 to median 11 (6–28) ng/ml after contrast application (p < 0.0001, Fig. 2). uNGAL at day 0 did not differ between patients who subsequently developed CI-AKI and those who did not (p = 0.76) (Table 2). A pro forma receiver operating characteristic (ROC) analysis gave an area under curve (AUC) of 0.51 (95% CI: 0.42–0.60), (Fig. 3). The threshold of uNGAL at day 0 that would have identified 20% patients as eligible for randomization as had been expected would have needed to be only 39 ng/ml. This corresponded to a sensitivity of only 28% (95% CI: 17–41%), a specificity of 80% (95% CI: 77–83%), and a positive predictive value of 10% (95% CI: 0.253–44.5%).

The relative changes of absolute uNGAL defined by the quotient of uNGAL at day 0 divided by day-1 were significantly correlated with the relative changes of the urine osmolality (Spearman r = 0.46, p < 0.0001), but this association was attenuated using uNGAL normalized to creatinine (Spearman r = 0.09, p = 0.03).

## Discussion

The main findings of this prospective randomized study of 617 patients with CKD undergoing intra-arterial angiography was that only 10 patients exhibited a significant rise of uNGAL exceeding the threshold for randomization and only one single patient out of these developed CI-AKI. Therefore, insufficient patients could be recruited to test the hypothesis that post-procedural volume expansion ameliorates CI-AKI. This was the first study of contrast induced nephropathy to attempt to triage patients to an intervention following the elevation of a kidney injury biomarker. Our findings contrast CI-AKI-studies in CKD-patients showing a good predictive power of uNGAL in the diagnosis of CI-AKI with a marked increase between six to twelve hours post angiography^4^,^25^,^26^. On the other hand, there are also observational studies demonstrating that in patients with an eGFR <60 ml/min uNGAL-levels did not differ between those with and without AKI until 24 hours after cardiac surgery^10^ and intra-arterial angiography^27^,^28^, respectively. A very recently published study of patients with an eGFR <30 ml/min revealed that NGAL was a reliable marker for ruling out CI-AKI but with a poor positive predictive power at 6 hours after CM-exposure^29^. A key conclusion from these studies as well as ours is that in CKD-patients uNGAL does not provide an early intervention strategy following contrast media application to prevent further kidney damage and a decline of kidney function. Two early intervention clinical trial paradigms have been proposed: (1) An early intervention after a renal insult or (2) an intervention after an early detection of a kidney injury prior to an observed change in kidney function^6^. Our results suggest that uNGAL is an inadequate tool for the latter strategy after contrast application. In general there is a great ambiguity of results inherent to studies on urinary and serum biomarkers for the diagnosis of AKI^30^ and this also seems to hold true for NGAL^31^. The diagnostic performance of NGAL is critically determined by baseline renal function and after a renal insult NGAL-levels in CKD-patients increase with a considerable delay^10^,^32^. Following cardiopulmonary bypass surgery, McIlroy and colleagues noted poor performance of NGAL in CKD patients at all time points following surgery^10^. In critically ill patients in the ICU Endre and colleagues noted that it was not until some 12 to 36 hours following the putative time of insult that the diagnostic performance of NGAL was optimized in this group^32^. If the findings in surgical and critically ill patients were to translate to the current setting, then this may explain the poor performance of NGAL as a tool to triage to early treatment. This clearly hampers a clinical decisive time advantage for an early diagnosis of AKI and the possibility of an early therapeutic intervention in CKD-patients. From ten randomized patients according to a relevant NGAL increase, only one patient developed a CI-AKI, whereas nine patients with a significant rise of uNGAL did not experience any subsequent deterioration of glomerular filtration rate. Due to the small number of randomized patients no meaningful conclusions about the benefits of additional intravenous volume expansion can be drawn.

In the whole cohort, baseline uNGAL-levels were significantly associated with diabetes, lower eGFR, Cystatin C, proteinuria, age and female sex. These associations are in line with previous studies and thus support the validity of our measurements^33^,^34^. On the other hand, these findings also underline that NGAL concentrations are associated with various parameters with relevance for CIN and kidney function thus questioning or limiting the additional prognostic and diagnostic value of uNGAL^35^. Baseline urine osmolality and its longitudinal changes were positively correlated with baseline absolute uNGAL concentrations and its relative changes. This finding favors the preferential use of the ratio to creatinine, thus minimizing distortions by fluctuating urine osmolality^36^,^14^,^37^. Normalization of urinary biomarkers to creatinine, however, is also under debate as creatinine secretion shows a high variability during changes in glomerular filtration rate^38^. In patients with normal kidney function a cut off NGAL-value >150 ng/ml seems to be suitable for the diagnosis of AKI^7^. CKD-patients exhibit higher NGAL concentrations at baseline and higher cut-off levels for the identification of evolving AKI are therefore presumed^39^. Taking the high biological variability of NGAL into account, the cut off levels in our study seem to be thoroughly appropriate for a CKD-population^14^,^15^,^37^. Surprisingly, in 70% of our patients the concentrations of both absolute and uNGAL normalized to creatinine dropped after contrast application with a more pronounced decrease of absolute uNGAL concentrations. In parallel urine osmolality also decreased, albeit to a much lesser extent suggesting that a diluting effect is probably involved. Consistent with our results an observational study showed that half of the patients exhibited a decrease of both absolute and uNGAL normalized to creatinine concentrations after coronary angiography^40^. The incidence of CI-AKI in our study was 9.4% and at the lower end of the range reported in the literature that is characterized by a great variability of results^1^. In more recent studies, frequencies of CI-AKI in CKD patients varied from 3.2% to 12.3%^4^,^25^,^26^,^27^,^29^ and our findings are in good agreement with that. As a limitation of these studies as well as ours it cannot be fully excluded that CI-AKI cases occurring beyond 48 hours after contrast application might have been missed. In contrast to previous studies, established and recognized risk factors for CI-AKI such as diabetes, age, proteinuria, contrast volume and renal impairment could not be identified as independent predictors of CI-AKI by logistic regression analysis in our cohort. Quite contrary, baseline renal function determined by plasma creatinine and eGFR but not by Cystatin C was better in patients developing a CI-AKI. A possible explanation for this surprising finding might be the regression to the mean phenomenon, especially as Cystatin C did not show to have any predictive significance. The occurrence of CI-AKI necessitated a longer hospital stay, but after one as well as after three months after hospital discharge, there was no significant difference in renal function between CI-AKI and non-CI-AKI patients. These results suggest that in our study, contrast media caused only minor kidney damage with only a transient deterioration of glomerular filtration rate.

Our study has some limitations. As it is a single-center study our data need confirmation in a multi-center study setting. In order to test our hypothesis, we did not provide intravenous fluid post contrast application to all our patients as current CI-AKI prevention guidelines recommend^5^. Indeed, the comparably low incidence of CI-AKI in our high risk cohort demonstrated that we did not harm our patients and pre-hydration alone might be sufficient enough to prevent CI-AKI. However, this finding has to be confirmed by appropriate studies. The aim of the study was to evaluate uNGAL as a triage tool and for that reason serial uNGAL-measurements were not performed. We therefore cannot provide sufficient information on uNGAL-kinetics in CKD-patients. On the other hand, to the best of our knowledge the ANTI-CI-AKI-study is the largest prospective randomized trial so far evaluating the diagnostic performance of uNGAL in identifying evolving CIN and an early intervention strategy in CKD-patients. The results of our study, along with the aforementioned negative biomarker studies, demonstrate that in order to be a successful triage tool the correct biomarker for a specific kidney injury measured at the correct time point has to be selected. We know of no study that has measured multiple biomarkers at multiple time points following contrast administration to provide guidance as to what may be the optimal biomarker in this setting.

In conclusion, in this study uNGAL failed to guide an early intervention strategy post contrast application and failed to identify evolving CI-AKI in CKD-patients after intra-arterial angiography. No conclusions can be drawn about the benefits of additional intravenous volume expansion post angiography in this cohort.

## Additional Information

**How to cite this article:** Ribitsch, W. *et al*. Neutrophil gelatinase-associated lipocalin (NGAL) fails as an early predictor of contrast induced nephropathy in chronic kidney disease (ANTI-CI-AKI study). *Sci. Rep.* **7**, 41300; doi: 10.1038/srep41300 (2017).

**Publisher's note:** Springer Nature remains neutral with regard to jurisdictional claims in published maps and institutional affiliations.

## Supplementary Material

Supplementary Information

## Figures and Tables

**Table 1:** Baseline characteristics and demographic data of all patients (*n* = 617).

Variable	Mean/median (±SD/Q_1_–Q_3_)
Age, y	74.0 (68–80)
Female, n (%)	287 (46.5)
Body mass index, kg/m^2^	27.2 ± 4.2
Hypertension, n (%)	530 (85.9)
Diabetes, n (%)	246 (39.9)
Heart disease, n	501 (81.2)
Baseline MAP, mmHg	97 (89–110)
Coronary angiography, n (%)	385 (62.4)
Peripheral vascular angiography, n (%)	213 (34.5)
Other localization, n (%)	19 (3.1)
Contrast volume, mL	100 (80–140)
NSAID, n (%)	23 (3.73)
RAAS-blocker, n (%)	473 (76.7)
Diuretics, n (%)	390 (63.2)
Serum creatinine, mg/dl	1.36 ± 0.41
eGFR (MDRD), ml·min^−1^·1.73 m^−2^	48.68 ± 12.66
Cystatin C (mg/L)	1.28 ± 0.45
Osmolality_urine_ (mOsm/kgH_2_O)	470 (370–610)
Urinary protein (mg/g Creatinine)	100 (69–190)
uNGAL (ng/ml), day-1	19 (9–49)
uNGAL (ng/ml), day0	11 (6–28)
uNGAL (μg/g Creatinine), day-1	25 (12–58)
uNGAL (μg/gCrea), day 0	20 (11–47)
Hospital stay (days)	2 (2–5)

*MAP*: mean arterial pressure, *NSAID*: nonsteroidal anti-inflammatory drugs, *RAAS*: renin angiotensin aldosterone system, *eGFR*: estimated glomerular filtration rate *(MDRD*: Modification of Diet in Renal Disease).

**Table 2:** Univariate predictors of CI-AKI derived by logistic regression analysis.

Variable	Patients without CIN (n = 559)Mean/median (±SD/Q1–Q_3_)	Patients with CIN (n = 58)Mean/median (±SD/Q1–Q_3_)	OR (95%-CI)	*p*-value
Age, y	74 (68–80)	76 (71–80)	1.03 (0.99–1.06)	0.13
BMI, kg/m^2^	27.25 ± 4.25	26.71 ± 3.44	0.97 (0.90–1.03)	0.31
MAP_day-1_, mmHg	97.48 ± 13.22	96.26 ± 13.75	0.99 (0.97–1.01)	0.46
Contrast volume, ml	110.12 ± 51.74	118.90 ± 54.83	1.00 (0.99–1.01)	0.18
eGFR_day-1_, ml·min^−1^·1.73m^−2^	48.68 ± 12.66	53.12 ±10.05	1.03 (1.01–1.06)	0.006
Se-Creatinine_day-1_, mg/dl	1.36 ± 0.41	1.19 ± 0.27	0.2 (0.07–0.50)	0.001
Cystatin C_day-1_, mg/L,	1.28 ± 0.45	1.21 ± 0.38	0.67 (0.32–1.28)	0.26
Urine osmolality_day-1_, mOsm/kgH_2_O	470 (370–610)	460 (370–580)	0.99 (0.99–1.00)	0.46
Urinary protein_day-1_, mg/gCreatinine	100 (69–190)	110 (77–170)	1.00 (0.99–1.00)	0.89
uNGAL (ng/ml)_day-1_	19 (9–49)	18 (9.2–48)	0.96 (0.58–1.58)	0.89
uNGAL (μg/gCrea)_day-1_	25 (12–57)	24 (13–76)	1.11 (0.67–1.83)	0.68
uNGAL (ng/ml)_day0_	11 (6–27)	13 (5–40)	1.04 (0.64–1.7)	0.76
uNGAL (μg/gCrea)_day0_	20 (10–44)	20 (11–57)	1.07 (0.65–1.8)	0.54
Sex (n, %)				
Female	256 (45.8)	31 (53.4)		
Male	303 (54.2)	27 (46.6)	0.74 (0.43–1.26)	0.27
Diabetes (n, %)				
No	330 (59)	41 (70.7)		
Yes	229 (41)	17 (29.3)	0.60 (0.32–1.06)	0.09
Heart disease (n, %)				
No	108 (19.3)	8 (13.8)		
Yes	451 (80.7)	50 (86.2)	1.5 (0.73–3.50)	0.31
Hypertension (n, %)				
No	80 (14.3)	7 (12.1)		
Yes	479 (85.7)	51 (87.9)	1.22 (0.57–3.00)	0.64
Diuretics (n, %)				
No	207 (37)	20 (34.5)		
Yes	352 (63)	38 (65.5)	1.12 (0.64–2.00)	0.7
RAAS-Blocker (n, %)				
No	131 (23.4)	13 (22.4)		
Yes	428 (76.6)	45 (77.6)	1.06 (0.57–2.10)	0.86
Statins (n, %)				
No	99 (37.4)	7 (25.0)		
Yes	166 (62.6)	21 (75.0)	1.79 (0.77–4.7)	0.2
Angiography (n, %)				
Coronary	385 (62.4)	42 (72.4)		
Peripheral arteries	213 (34.5	14 (24.1)	0.57 (0.3–1.1)	0.08
other	19 (3.08)	2 (3.45)	0.96 (0.15–3.50)	0.96

OR = odds ratio, CI = confidence interval.

**Table 3:** Association of absolute baseline uNGAL and uNGAL normalized to creatinine with clinical variables.

Variable	Baseline uNGAL (ng/ml)Median (95%-CI)	*p*-value	Baseline uNGAL (μg/g Creatinine)Median (95%-CI)	*p*-value
Sex				
Female	31 (25–36)	<0.0001	40 (33–46)	<0.0001
Male	16 (13–18)		19 (18–21)	
Age, years				
≤74	17 (15–22)	0.009	21 (18–24)	<0.0001
>74	22 (19–27)		30 (25–35)	
BMI, kg/m^2^				
≤27	20 (17–24)	0.94	25 (22–29)	0.66
>27	19 (16–24)		24 (20–29)	
Diabetes				
yes	24 (18–31)	0.002	30 (26–37)	0.0002
No	18 (15–22)		22 (20–25)	
Hypertension				
yes	19 (17–23)	0.86	25 (22–28)	0.38
No	23 (14–29)		22 (18–28)	
Heart disease				
yes	18 (16–22)	0.005	24 (22–26)	0.03
No	29 (20–36)		30 (23–42)	
Se-Creatinine (mg/dl), day-1				
≤1.2	19 (16–24)	0.57	24 (21–28)	0.38
>1.2	20 (17–24)		26 (22–29)	
eGFR (ml·min^−1^·1.73 m^−2^), day-1:			21 (17–26)	
>60	18 (14–24)	0.0002	21 (18–24)	<0.0001
59–45	17 (13–22)		30 (27–39)	
44–30	22 (18–27)		49 (34–72)	
29–15	44 (28–54)		20 (20–20)	
<15	7 (7–7)			
Cystatin C (mg/L), day-1				
≤1.2	18 (15–22)	0.001	21 (18–23)	<0.0001
>1.2	22 (18–27)		33 (27–37)	
Osmolality_urine_ (mOsm/kgH_2_O), day-1				
≤470	14 (12–17)	<0.0001	30 (26–36)	<0.0001
>470	26 (22–30)		21 (19–23)	
Urinary protein (mg/gCreatinine) day-1				
≤100	16 (13–18)	<0.0001	18 (16–20)	<0.0001
>100	27 (22–31)		37 (33–44)	
Hospital stay (days)				
≤2	20 (17–24)	0.54	26 (24–29)	0.42
>2	18 (16–24)		22 (20–28)	

*P*-values were derived from Kruskal-Wallis test. Continuous variables were grouped into above and below the median with 95% confidence intervals in parenthesis.
