# Supplementary material for: Neutrophil gelatinase-associated lipocalin (NGAL) fails as an early predictor of contrast induced nephropathy in chronic kidney disease (ANTI-CI-AKI study)
Source: Sci Rep. 2017 Jan 27;7:41300. doi: 10.1038/srep41300 (PMC5269674; doi:10.1038/srep41300)
Supplement: Supplementary Information [file srep41300-s1.pdf]

Neutrophil gelatinase-associated lipocalin (NGAL) fails as an early predictor of contrast induced nephropathy in chronic kidney disease (ANTI-CI-AKI study)

Werner Ribitsch[1], Gernot Schilcher[1,2*], Franz Quehenberger[3], Stefan Pilz[4], Rupert H Portugaller[5], Martini Truschnig-Wilders [6], Robert Zweiker[7], Marianne Brodmann[8], Philipp Stiegler[9], Alexander R Rosenkranz[1], John W Pickering[10] and Joerg H Horina[1]

[1]Clinical Division of Nephrology, Department of Internal Medicine, Medical University of Graz (MUG), Austria
[2]Intensive Care Unit, Department of Internal Medicine, MUG, Austria
[3]Institute for Medical Informatics, Statistics and Documentation, Medical University of Graz, Austria
[4]Division of Endocrinology and Metabolism, Department of Internal Medicine, Medical University of Graz, Austria
[5]Department of Vascular and Interventional Radiology, University Clinic of Radiology, Medical University of Graz, Austria
[6]Clinical Institute for Medical and Chemical Laboratory Diagnostics, Medical University of Graz, Austria
[7]Division of Cardiology, Department of Internal Medicine, Medical University of Graz, Austria
[8]Division of Angiology, Department of Internal Medicine, Medical University of Graz, Austria
[9]Division of Transplantation Surgery, Medical University of Graz, Austria
[10]Department of Medicine, University of Otago Christchurch and Emergency Medicine Department, Christchurch Hospital, Christchurch, New Zealand

Supplementary Appendix

Supplemental Table 1. Adverse events (AE) during the ANTI-CIN -study

**Adverse event (*n*)**	**AE Day -1**	**SAE Day-1**	**AE Day 0**	**SAE Day 0**	**AE Day 1**	**SAE Day 1**	**AE Day 2**	**SAE Day 2**
Pain (*localization)*								
Headache	7		8		6		2	
Musculosceletal system	20		69		25		20	1
Thorax	1		4	3	1			
Abdomen	1		4		1		1	
Cardiac (Arrhythmias, NSTEMI, pulmonary edema,hypotension)		4	27	6	7	4	1	
Hypertensive crisis	8		45		9			
Neurological (Dizziness, mental confusion,	6	1	5	4	11	1	2	2
Nausea, vomiting	2	3	11		6		3	
Dyspnoea			7		6		2	
Epistaxis	1	1						
Infection	1	4	2	1	4		2	2
Dermatological (rash, exanthema, pruritus)	1		16		10			1
Peri-interventional complications								
Dissection								
Bradycardia			5	30				
Tachycardia			3					
Hypotension			5					
Hypertension			19					
Hematoma			10					
Embolism			2					
Death				1				
Other			13					
Total	48	13	255	45	86	5	33	6

Supplemental Table 2. Adverse reactions (AR) during the ANTI-CIN -study

**Adverse reaction (*n*)**	**AR Day -1**	**SAR Day-1**	**AR Day 0**
Headache	1		
Cardial<br>(Arrhythmias, NSTEMI, pulmonary edema,hypotension)	1		
Hypertensive crisis		2	1
Dyspnoea	6		
Neurological	1		
Total	9	2	1
